# Supplementary material for: MetaRibo-Seq measures translation in microbiomes
Source: Nat Commun. 2020 Jun 29;11:3268. doi: 10.1038/s41467-020-17081-z (PMC7324362; doi:10.1038/s41467-020-17081-z)
Supplement: Supplementary file 10 — Supplementary Data 7 [file 41467_2020_17081_MOESM10_ESM.zip › File2/Confidence_High_Taxonomy/290633_out.krona.html]

Javascript must be enabled to view this page.

members
magnitude
magnitudeUnassigned
count
unassigned
taxon
rank

290633\_out

422

422
superkingdom
2

976
phylum
422

200643
class
422

422
171549
order

2
family
171552

577309
genus
1

454154

SRS015782\_contig\_number\_46863
species
1

1
838
genus

1
species

SRS044535\_contig\_number\_14732
165179

420
family
815

816
genus
420

species

SRS024625\_contig\_number\_8159
2292002
1

2
671267
species

SRS013687\_contig\_number\_28111SRS147271\_contig\_number\_24906

2292943

SRS024435\_contig\_number\_31173
species
1

1
2292950
species

SRS019267\_contig\_number\_4674

4

SRS023715\_contig\_number\_20528SRS051031\_contig\_number\_34460SRS1041144\_contig\_number\_1475SRS893172\_contig\_number\_14353
species
46506

1

SRS018133\_contig\_number\_2816
species
1638779

371601
species

SRS024075\_contig\_number\_9318SRS053214\_contig\_number\_6474
2

2292944

SRS043768\_contig\_number\_16709SRS971275\_contig\_number\_31661
species
2

820

SRS014412\_contig\_number\_18298
species
1

12
species

SRS013951\_contig\_number\_41156SRS015890\_contig\_number\_1449SRS016018\_contig\_number\_11866SRS056519\_contig\_number\_3953SRS057717\_contig\_number\_1477SRS058070\_contig\_number\_24331SRS077194\_contig\_number\_5009SRS098881\_contig\_number\_13751SRS1055022\_contig\_number\_11857SRS142503\_contig\_number\_3312SRS145308\_contig\_number\_470SRS149075\_contig\_number\_18548
818

357276

SRS018049\_contig\_number\_1126SRS018575\_contig\_number\_7546SRS019685\_contig\_number\_33512SRS023346\_contig\_number\_6891SRS042628\_contig\_number\_27127SRS043001\_contig\_number\_3471SRS045195\_contig\_number\_4890SRS052078\_contig\_number\_924SRS054590\_contig\_number\_421SRS058770\_contig\_number\_15060SRS075341\_contig\_number\_22952SRS077024\_contig\_number\_12864SRS098655\_contig\_number\_11784SRS098717\_contig\_number\_18783SRS101268\_contig\_number\_207SRS1055034\_contig\_number\_9890SRS140645\_contig\_number\_1233SRS142712\_contig\_number\_1894SRS148874\_contig\_number\_5434SRS149784\_contig\_number\_28385
species
20

4
28116

SRS013476\_contig\_number\_34931SRS019030\_contig\_number\_12355SRS104084\_contig\_number\_12794SRS143372\_contig\_number\_7643
species


SRS015694\_contig\_number\_16640SRS1054716\_contig\_number\_14632
species
338188
2

1

SRS017821\_contig\_number\_19058
species
2292941

821

SRS011084\_contig\_number\_32884SRS011239\_contig\_number\_22784SRS011302\_contig\_number\_27862SRS011405\_contig\_number\_11671SRS012849\_contig\_number\_27008SRS012902\_contig\_number\_96SRS012969\_contig\_number\_34977SRS013098\_contig\_number\_50210SRS013158\_contig\_number\_969SRS013382\_contig\_number\_2347SRS013521\_contig\_number\_8787SRS013638\_contig\_number\_11684SRS013800\_contig\_number\_12801SRS013965\_contig\_number\_41860SRS014235\_contig\_number\_41159SRS014459\_contig\_number\_8230SRS014534\_contig\_number\_671SRS014683\_contig\_number\_16896SRS014736\_contig\_number\_1803SRS014855\_contig\_number\_11168SRS014948\_contig\_number\_23783SRS014979\_contig\_number\_22513SRS015065\_contig\_number\_17036SRS015190\_contig\_number\_14151SRS015217\_contig\_number\_30292SRS015264\_contig\_number\_17594SRS015369\_contig\_number\_8404SRS015431\_contig\_number\_20879SRS015486\_contig\_number\_332SRS015578\_contig\_number\_28506SRS015579\_contig\_number\_17508SRS015663\_contig\_number\_31069SRS015794\_contig\_number\_12650SRS015854\_contig\_number\_6657SRS015960\_contig\_number\_17710SRS016056\_contig\_number\_304SRS016132\_contig\_number\_4111SRS016203\_contig\_number\_9222SRS016335\_contig\_number\_14077SRS016381\_contig\_number\_contig-100\_3.34841SRS016495\_contig\_number\_19191SRS016629\_contig\_number\_contig-100\_2607.30476SRS016753\_contig\_number\_626SRS016840\_contig\_number\_2729SRS016897\_contig\_number\_312SRS016954\_contig\_number\_15160SRS017103\_contig\_number\_17388SRS017191\_contig\_number\_6150SRS017247\_contig\_number\_4878SRS017307\_contig\_number\_19348SRS017375\_contig\_number\_146SRS017433\_contig\_number\_10556SRS017701\_contig\_number\_10110SRS017745\_contig\_number\_2630SRS017859\_contig\_number\_3348SRS018351\_contig\_number\_17277SRS018427\_contig\_number\_30647SRS018623\_contig\_number\_15171SRS018817\_contig\_number\_21101SRS018836\_contig\_number\_27120SRS018888\_contig\_number\_9230SRS018936\_contig\_number\_12248SRS018984\_contig\_number\_4845SRS019068\_contig\_number\_38397SRS019161\_contig\_number\_7728SRS019178\_contig\_number\_5426SRS019381\_contig\_number\_5135SRS019397\_contig\_number\_1197SRS019445\_contig\_number\_27383SRS019496\_contig\_number\_14774SRS019601\_contig\_number\_30080SRS019693\_contig\_number\_9203SRS019808\_contig\_number\_20561SRS019910\_contig\_number\_10796SRS019968\_contig\_number\_13780SRS020233\_contig\_number\_48949SRS020328\_contig\_number\_23327SRS020394\_contig\_number\_15198SRS020508\_contig\_number\_1007SRS020622\_contig\_number\_675SRS020869\_contig\_number\_29744SRS021153\_contig\_number\_979SRS021219\_contig\_number\_1675SRS021484\_contig\_number\_26284SRS021948\_contig\_number\_17157SRS022071\_contig\_number\_33060SRS022137\_contig\_number\_18057SRS022524\_contig\_number\_16280SRS022713\_contig\_number\_273SRS023526\_contig\_number\_25784SRS023583\_contig\_number\_15334SRS023829\_contig\_number\_50SRS023914\_contig\_number\_13780SRS023971\_contig\_number\_3988SRS024009\_contig\_number\_10230SRS024132\_contig\_number\_37271SRS024331\_contig\_number\_18423SRS024492\_contig\_number\_15620SRS042284\_contig\_number\_4014SRS042966\_contig\_number\_11385SRS043411\_contig\_number\_13093SRS043667\_contig\_number\_5767SRS043701\_contig\_number\_10321SRS045004\_contig\_number\_2201SRS045244\_contig\_number\_2052SRS045645\_contig\_number\_14207SRS045739\_contig\_number\_2604SRS045826\_contig\_number\_12532SRS046369\_contig\_number\_15110SRS046502\_contig\_number\_12617SRS046810\_contig\_number\_contig-100\_12.13SRS047014\_contig\_number\_contig-100\_1835.182035SRS047044\_contig\_number\_16414SRS047433\_contig\_number\_12152SRS048164\_contig\_number\_24470SRS048870\_contig\_number\_18717SRS049402\_contig\_number\_9134SRS049446\_contig\_number\_10072SRS049896\_contig\_number\_1731SRS049900\_contig\_number\_14983SRS049900\_contig\_number\_19945SRS049959\_contig\_number\_37148SRS049995\_contig\_number\_9133SRS050026\_contig\_number\_3851SRS050299\_contig\_number\_5339SRS050422\_contig\_number\_37568SRS050520\_contig\_number\_contig-100\_36.21421SRS050752\_contig\_number\_24516SRS050801\_contig\_number\_contig-100\_677.28724SRS050941\_contig\_number\_17859SRS050974\_contig\_number\_contig-100\_5.24044SRS050998\_contig\_number\_13369SRS051610\_contig\_number\_contig-100\_288.69926SRS051882\_contig\_number\_16209SRS052576\_contig\_number\_contig-100\_11.15520SRS052697\_contig\_number\_19382SRS053087\_contig\_number\_2554SRS053335\_contig\_number\_18567SRS053356\_contig\_number\_47006SRS053398\_contig\_number\_15198SRS054059\_contig\_number\_5139SRS054352\_contig\_number\_22911SRS054905\_contig\_number\_4428SRS055017\_contig\_number\_16737SRS055966\_contig\_number\_66SRS055982\_contig\_number\_12828SRS056259\_contig\_number\_13101SRS056537\_contig\_number\_26287SRS057478\_contig\_number\_2501SRS057480\_contig\_number\_10298SRS058145\_contig\_number\_contig-100\_3560.3561SRS058268\_contig\_number\_3SRS058723\_contig\_number\_1061SRS062427\_contig\_number\_7655SRS062654\_contig\_number\_12634SRS062701\_contig\_number\_5170SRS063040\_contig\_number\_802SRS063127\_contig\_number\_1899SRS063190\_contig\_number\_3977SRS063370\_contig\_number\_3233SRS063489\_contig\_number\_2140SRS063518\_contig\_number\_15617SRS063813\_contig\_number\_5294SRS063928\_contig\_number\_475SRS063985\_contig\_number\_30457SRS064557\_contig\_number\_4288SRS064645\_contig\_number\_794SRS064757\_contig\_number\_23960SRS064973\_contig\_number\_2045SRS065176\_contig\_number\_10197SRS065397\_contig\_number\_12154SRS065504\_contig\_number\_25307SRS074586\_contig\_number\_5471SRS074670\_contig\_number\_2426SRS074964\_contig\_number\_1723SRS075398\_contig\_number\_280SRS075716\_contig\_number\_3969SRS075773\_contig\_number\_36114SRS075821\_contig\_number\_1992SRS075878\_contig\_number\_21648SRS075984\_contig\_number\_18073SRS076756\_contig\_number\_14066SRS076876\_contig\_number\_420SRS076976\_contig\_number\_16213SRS077086\_contig\_number\_10997SRS077127\_contig\_number\_13648SRS077231\_contig\_number\_22781SRS077294\_contig\_number\_16441SRS077335\_contig\_number\_5698SRS077392\_contig\_number\_42728SRS077454\_contig\_number\_17848SRS077502\_contig\_number\_6382SRS077552\_contig\_number\_25359SRS077589\_contig\_number\_1545SRS077641\_contig\_number\_21394SRS077730\_contig\_number\_4471SRS077849\_contig\_number\_18405SRS077991\_contig\_number\_249SRS078176\_contig\_number\_21197SRS078242\_contig\_number\_28290SRS078419\_contig\_number\_21088SRS078665\_contig\_number\_7329SRS097889\_contig\_number\_4584SRS097920\_contig\_number\_15391SRS097958\_contig\_number\_14579SRS098061\_contig\_number\_1244SRS098073\_contig\_number\_13884SRS098571\_contig\_number\_55627SRS098644\_contig\_number\_35489SRS098827\_contig\_number\_322SRS100021\_contig\_number\_2215SRS100907\_contig\_number\_contig-100\_48.68584SRS101376\_contig\_number\_22771SRS101433\_contig\_number\_8793SRS103987\_contig\_number\_22021SRS104036\_contig\_number\_contig-100\_1395.54765SRS1041031\_contig\_number\_10509SRS1041032\_contig\_number\_14742SRS1041036\_contig\_number\_2253SRS1041037\_contig\_number\_15966SRS1041038\_contig\_number\_16517SRS1041039\_contig\_number\_28131SRS1041091\_contig\_number\_22493SRS1041092\_contig\_number\_8378SRS1041095\_contig\_number\_4961SRS1041116\_contig\_number\_7321SRS1041118\_contig\_number\_14822SRS1041129\_contig\_number\_6542SRS1041130\_contig\_number\_1601SRS1041133\_contig\_number\_25048SRS1041134\_contig\_number\_14835SRS1041136\_contig\_number\_16662SRS1041137\_contig\_number\_29462SRS1041138\_contig\_number\_3186SRS1041139\_contig\_number\_1565SRS1041140\_contig\_number\_23189SRS1041142\_contig\_number\_4177SRS1041143\_contig\_number\_4158SRS1041147\_contig\_number\_1107SRS1041157\_contig\_number\_5555SRS1041164\_contig\_number\_1348SRS104327\_contig\_number\_3130SRS104400\_contig\_number\_3643SRS104485\_contig\_number\_66SRS104693\_contig\_number\_4519SRS104912\_contig\_number\_17372SRS104975\_contig\_number\_19691SRS105082\_contig\_number\_20687SRS105153\_contig\_number\_14964SRS1054691\_contig\_number\_16293SRS1054928\_contig\_number\_4251SRS1055038\_contig\_number\_3891SRS1055056\_contig\_number\_13176SRS1055067\_contig\_number\_9965SRS1055069\_contig\_number\_10378SRS1055076\_contig\_number\_6802SRS1055083\_contig\_number\_4288SRS1055095\_contig\_number\_993SRS1055099\_contig\_number\_13684SRS1055099\_contig\_number\_13734SRS140492\_contig\_number\_3199SRS140513\_contig\_number\_5492SRS142599\_contig\_number\_26559SRS142781\_contig\_number\_5254SRS142890\_contig\_number\_21050SRS142980\_contig\_number\_953SRS143070\_contig\_number\_16025SRS143085\_contig\_number\_1504SRS143148\_contig\_number\_13567SRS143342\_contig\_number\_5543SRS143417\_contig\_number\_16883SRS143523\_contig\_number\_20999SRS143780\_contig\_number\_22057SRS143876\_contig\_number\_11384SRS143895\_contig\_number\_contig-100\_9.258333SRS143991\_contig\_number\_18069SRS144135\_contig\_number\_35904SRS144297\_contig\_number\_12353SRS144362\_contig\_number\_6353SRS144506\_contig\_number\_47836SRS144537\_contig\_number\_32128SRS144603\_contig\_number\_13728SRS144714\_contig\_number\_22247SRS144753\_contig\_number\_contig-100\_44.87405SRS145497\_contig\_number\_4740SRS146812\_contig\_number\_54853SRS146813\_contig\_number\_28885SRS147022\_contig\_number\_1349SRS147039\_contig\_number\_4112SRS147271\_contig\_number\_33110SRS147346\_contig\_number\_6073SRS147377\_contig\_number\_9079SRS147425\_contig\_number\_10910SRS147557\_contig\_number\_34507SRS147614\_contig\_number\_7712SRS147766\_contig\_number\_19159SRS147977\_contig\_number\_8675SRS148091\_contig\_number\_14589SRS148159\_contig\_number\_28435SRS148196\_contig\_number\_41287SRS148253\_contig\_number\_14324SRS148319\_contig\_number\_contig-100\_2459.89486SRS148511\_contig\_number\_18626SRS148721\_contig\_number\_53617SRS148784\_contig\_number\_26103SRS148817\_contig\_number\_370SRS148970\_contig\_number\_1305SRS149181\_contig\_number\_31476SRS150029\_contig\_number\_14645SRS892882\_contig\_number\_16054SRS893170\_contig\_number\_2365SRS893187\_contig\_number\_13761SRS893230\_contig\_number\_10971SRS893252\_contig\_number\_5382SRS893253\_contig\_number\_70SRS893256\_contig\_number\_278SRS893259\_contig\_number\_5960SRS893270\_contig\_number\_24754SRS893288\_contig\_number\_3969SRS893292\_contig\_number\_6776SRS893295\_contig\_number\_10540SRS893341\_contig\_number\_9882SRS893342\_contig\_number\_5591SRS893358\_contig\_number\_11627SRS893366\_contig\_number\_contig-100\_733.145170SRS893369\_contig\_number\_8546SRS893373\_contig\_number\_20538SRS893378\_contig\_number\_3491SRS893380\_contig\_number\_316SRS893382\_contig\_number\_5574SRS893383\_contig\_number\_7443SRS971276\_contig\_number\_17862
species
332

1

SRS143722\_contig\_number\_9282
species
457387


SRS013215\_contig\_number\_145SRS024549\_contig\_number\_2546SRS024663\_contig\_number\_1765
species
457395
3

species

SRS053649\_contig\_number\_3405SRS142542\_contig\_number\_15566
1263054
2

1739517

SRS046068\_contig\_number\_463
species
1

457391

SRS018541\_contig\_number\_9336SRS1041112\_contig\_number\_318SRS1055043\_contig\_number\_12964
species
3

329854

SRS142923\_contig\_number\_19591
species
1


SRS049712\_contig\_number\_989SRS147653\_contig\_number\_255SRS149325\_contig\_number\_10554SRS893285\_contig\_number\_230
species
310297
4

28111
species

SRS075021\_contig\_number\_1372
1


SRS014613\_contig\_number\_contig-100\_96.123796SRS048060\_contig\_number\_8097SRS064232\_contig\_number\_3378SRS064276\_contig\_number\_41436SRS1041145\_contig\_number\_28726
species
2292935
5

4
246787

SRS014287\_contig\_number\_14494SRS045713\_contig\_number\_16492SRS1041141\_contig\_number\_9660SRS148424\_contig\_number\_20087
species

2
1263042

SRS012273\_contig\_number\_11986SRS013940\_contig\_number\_22514
species

3
47678
species

SRS014313\_contig\_number\_416SRS049773\_contig\_number\_3647SRS143598\_contig\_number\_18442

4
817
species

SRS016095\_contig\_number\_17335SRS048981\_contig\_number\_4327SRS146764\_contig\_number\_22404SRS893279\_contig\_number\_6788
